# Effects of Kimchi-Derived Lactic Acid Bacteria on Reducing Biological Hazards in Kimchi

**DOI:** 10.4014/jmb.2408.08016

**Published:** 2024-10-25

**Authors:** Yeonsoo Shim, Jae Yong Lee, Jihye Jung

**Affiliations:** Industrial Solution Research Group, World Institute of Kimchi, Gwangju 61755, Republic of Korea

**Keywords:** Lactic acid bacteria, kimchi, antimicrobial activity, foodborne pathogen, soft-rot pathogen, kimchi cabbage

## Abstract

This study was performed to investigate the use of plant-based lactic acid bacteria (LAB) to reduce microbiological hazards in kimchi. Cell-free supernatants (CFS) from four LAB strains isolated from kimchi were tested for antimicrobial activity against five foodborne pathogens and two soft-rot pathogens. Each CFS showed antimicrobial activity against both foodborne and soft-rot pathogens. Washing salted kimchi cabbages inoculated with *B. cereus* with 5% CFS inhibited *B. cereus* to a greater extent than NaClO. The CFS from WiKim 83 and WiKim 87 exhibits inhibition rates of 25.09% and 24.21%, respectively, compared to the 19.19% rate of NaClO. Additionally, the CFS from WiKim 116 and WiKim 117 showed inhibition rates of 18.74% and 20.03%, respectively. Direct treatment of kimchi cabbage with soft-rot pathogens and CFS for five days inhibited the pathogens with similar efficacy to that of NaClO. To elucidate the antimicrobial activity mechanisms, pH neutralization, heat treatment, and organic acid analyses were performed. pH neutralization reduced the antimicrobial activity, whereas heat treatment did not, indicating that lactic, acetic, citric, and phenyllactic acids contribute to the thermal stability and antimicrobial properties of CFS. This study suggests that the four kimchi-derived LAB, which maintain a low pH through organic acid production, could be viable food preservatives capable of reducing biological hazards in kimchi.

## Introduction

Kimchi, a staple of Korean cuisine enjoyed globally, faces growing concerns about its safety for both domestic and international consumption [1]. This is due to potential contamination risks that arise during the production and supply processes. Kimchi harbors various microorganisms during the fermentation process, some of which may be pathogenic or spoilage bacteria, particularly when contamination occurs during processing [2]. These microorganisms can act as biological hazards in kimchi, potentially causing foodborne illnesses. To ensure consumer safety, the Korean Ministry of Food and Drug Safety (MFDS) has established stringent microbiological specifications for kimchi. For example, pathogens such as *Salmonella* spp., *Vibrio parahaemolyticus*, *Listeria monocytogenes*, enterohemorrhagic *Escherichia coli* (EHEC), *Campylobacter jejuni/coli*, and *Yersinia enterocolitica* must be entirely absent across five samples (*n* = 5, *c* = 0, *m* = 0/25 g). For *Bacillus cereus*, the limit is 10,000 CFU/g for fermented foods like kimchi, sauces, and mixed seasonings. In addition, sterilized products must test negative for the pathogen. *Clostridium perfringens* has a limit of *n* = 5, *c* = 2, *m* = 100, *M* = 1,000 for products such as fermented foods, with stricter standards (*n* = 5, *c* = 0, *m* = 0/25 g) for sterilized items. Similarly, the limits for *Staphylococcus aureus* are *n* = 5, *c* = 0, *m* = 0/25 g. However, instances of kimchi failing these standards or causing illness outbreaks owing to pathogenic contamination have been reported, necessitating recalls and reinforcing the need for robust food safety measures [3-7].

The detected levels of foodborne pathogens in kimchi have repeatedly violated safety standards and resulted in outbreaks of foodborne illnesses. Despite the foodborne pathogen detection standards set by the MFDS [7], the detection of pathogens, such as *B. cereus*, *C. perfringens*, EHEC, *L. monocytogenes*, and *Y. enterocolitica* in kimchi, has been consistently reported. For example, in 2022, *C. perfringens* was detected in diced-radish kimchi (kkakdugi), and in 2011, both cabbage kimchi and diced-radish kimchi were found to contain *C. perfringens*, leading to product recalls [3]. In 2011, *L. monocytogenes* was detected in kimchi, halting its distribution and recall [4]. In 2012, an illness outbreak involving 1,200 students across seven schools in Gyeonggi Province was traced to EHEC found in patient stool samples [5]. In 2021, an outbreak of foodborne illness caused by *B. cereus* in Okcheon County was linked to contaminated cabbage kimchi. More recently, in 2023, *Y. enterocolitica* was detected in white kimchi, leading to a suspension of sales and subsequent recalls. Foodborne pathogens were also discovered in imported kimchi, raising concerns about the safety of these products [6]. These studies underscore the need for continuous efforts to control foodborne pathogens in kimchi and enhance food safety.

Kimchi cabbage is susceptible to various diseases such as soft rot caused by *Pectobacterium carotovorum*, which causes decay in major vegetable crops, including cabbage, by producing enzymes that degrade cell walls [8]. This bacterium infects cabbage in the soil and causes rot before harvest. Additionally, it can survive for an extended period after harvest, resulting in quality deterioration during storage, shortening the shelf-life of cabbage kimchi, and altering its texture [9]. This not only affects the quality of cabbage but also poses economic risks to kimchi producers [10, 11]. As kimchi typically does not undergo heat treatment during production, meticulous washing of raw ingredients, especially cabbage, is critical for control of pathogenic and spoilage microbes.

While NaClO is commonly used in kimchi production for washing raw materials, concerns over health risks associated with its excessive or improper use have prompted the exploration of safer alternatives [12]. The chemical preservatives traditionally used to control microbes in kimchi have several drawbacks, including potential antibiotic resistance among pathogens [13], allergic reactions [14], and environmental pollution. Thus, there is a growing interest in natural substances with antimicrobial properties, particularly those found in fermented foods. Recently, research on natural substances with antimicrobial properties for safe mitigation of microbiological hazards has garnered increasing interest. Studies with a focus on the application of antimicrobial activity derived from the metabolic byproducts of lactic acid bacteria found in fermented foods are gaining attention [15-17]. LAB contribute to food safety by producing antimicrobial substances, such as organic acids, hydrogen peroxide, and bacteriocins during fermentation [18, 19]. These compounds help to inhibit pathogenic microorganisms and spoilage bacteria, thereby maintaining food safety.

Lactic acid bacteria, generally regarded as safe (GRAS) by the FDA, produce organic acids, such as lactic acid, acetic acid, and phenyllactic acid, during fermentation. These organic acids lower the pH of kimchi, creating a hostile environment for pathogenic bacteria, while enhancing food safety [20], freshness, and sensory attributes [21]. Numerous studies have reported strong antibacterial and antifungal activities of *Lactiplantibacillus* and *Latilactobacillus* species [22-24]. Such research has generated interest in the potent antimicrobial properties of plant-based LAB derived from kimchi. Currently, the application in the food industry of CFS from LAB, including LAB metabolites, is being explored. CFS is anticipated to make significant contributions to the food industry owing to its high stability in various environments [25]. LAB strains isolated from kimchi not only establish stable microbial communities within kimchi but also suppress the proliferation of harmful bacteria through their metabolites. Moreover, the antimicrobial compounds present in the CFS are expected to control the microbial hazards of kimchi and enhance its safety.

Despite the ongoing efforts to control the harmful bacteria in kimchi, there is a notable lack of research on materials that can mitigate these hazards and their industrial applications. Therefore, studies focusing on reducing the numbers of pathogenic bacteria that cause kimchi spoilage and affect food hygiene and safety are crucial.

This study utilized plant-derived LAB isolated from kimchi to evaluate antimicrobial activities against foodborne pathogens and cabbage spoilage organisms. We aimed to highlight the potential of reducing the microbiological hazards in kimchi, while assessing the role of these LAB as indirect treatments in food washing and sterilization, and as direct preservatives in food applications.

## Materials and Methods

### Bacterial Strains and Culture Conditions

The bacterial strains used in this study and their culture conditions are listed in Table 1. Foodborne pathogens (*B. cereus* KCCM 40935, *L. monocytogenes* KCCM 40307, *E. coli* KCCM 11234, *C. perfringens* ATCC 13125, *Y. enterocolitica* ATCC 23715) were obtained from the Korean Culture Center of Microorganisms (KCCM) and the American Type Culture Collection (ATCC). Soft-rot pathogens (*P. carotovorum* KACC 10371, *P. carotovorum* KACC 10342) were obtained from the Korean Agricultural Culture Collection (KACC). Each pathogen was stored with tryptic soy broth (TSB; Difco, USA) containing 25% glycerol at -70°C in a deep freezer. Each foodborne or soft-rot pathogen was cultivated in 10 ml of TSB for 24 h.

The LAB strains (*Lactiplantibacillus plantarum* WiKim 83, *L. plantarum* WiKim 87, *Latilactobacillus sakei* WiKim 116, *L. sakei* WiKim 117) were provided by the World Institute of Kimchi. Each LAB strain was stored in De Man Rogosa Sharpe broth (MRS; Difco) containing 25% glycerol at -70°C in a deep freezer. Each LAB was grown anaerobically in 10 ml of MRS broth at 37°C for 24 h.

### Preparation of CFS

LAB was inoculated anaerobically in MRS broth at 37°C for 24 h. To normalize the CFS samples, the bacterial culture was prepared from LAB with a cell count confirmed to be over 10^9^ CFU/ml. To prepare the CFS, the culture was centrifuged at 13,870 ×*g* for 15 min at 4°C. After centrifugation, the supernatant was filtered through a 0.22-mm filter (Sartorius, Germany). CFS was used for further experiments.

### Antimicrobial Activity of CFS and Determination of Minimum Inhibitory Concentration (MIC) and Minimal Bactericidal Concentration (MBC)

The antimicrobial activity of CFS was evaluated using the dilution-in-broth method [26]. For this, 100 μl of each pathogen (10^6^ CFU/ml) was added to a 96-well microplate containing different concentrations of CFS (50, 25, 12.5, 6.25, and 3.12%) in a total volume of 100 μl with MRS. Uninoculated TSB was also prepared following the same protocol to generate a negative control. The plate was then incubated for 24 h, followed by a visual reading. The MIC was considered as the lowest dose at which no visible turbidity was observed due to the presence of bacteria. The MBC endpoint was defined as the lowest concentration of CFS that resulted in the elimination of the pathogens, as evidenced by the absence of visible bacterial growth on the TSA after incubation for 24 h.

### Time-Kill Assay for Evaluating *B. cereus* and *P. carotovorum* Inhibition

To evaluate the bactericidal effects of CFS against *B. cereus* and *P. carotovorum* over time, a modified time-kill assay was conducted [27]. *B. cereus* and *P. carotovorum* samples diluted to 10^4^ CFU/ml in TSB were exposed to CFS and 100 ppm NaClO at a 1:1 ratio relative to the 500 μl of bacterial suspension at intervals, followed by incubation on TSA plates at 30°C and 28°C for 24 h to enumerate viable counts. As a positive control, the pathogen was treated with 100 ppm NaClO.

### Biocontrol Assay for Evaluating *B. cereus* Inhibition Using CFS in Salted Kimchi Cabbage

Experiments were conducted using NaClO and CFS to control *B. cereus* in salted kimchi cabbage. Approximately 100 g of salted kimchi cabbage inoculated with *B. cereus* (10^4^ CFU/g) was dried for 30 min and used as the experimental sample. These samples were immersed in distilled water containing 100 ppm NaClO and 5% CFS, as per the guidelines of the MFDS [28]. After 5 min, a quantitative analysis of *B. cereus* was performed according to the Food Code.

### Biocontrol Assay to Investigate Soft-Rot Disease Bacteria Using CFS on Kimchi Cabbage

Experiments were conducted to investigate the control of soft rot in kimchi cabbage using NaClO and CFS. Kimchi cabbage samples, sized 4.0 × 4.0 cm, were washed with 100 ppm NaClO before use in the experiment. The samples were then inoculated with bacterial soft-rot at a concentration of 10^4^ CFU/ml, treated with CFS as well as a positive control of 100 ppm NaClO, and a negative control of distilled water. After inoculation with 10 μl of the suspension, the kimchi cabbage samples were air-dried at room temperature for 30 min. Subsequently, the inoculated samples were cultured at 25°C for 5 days to observe disease progression.

### Effects of pH Neutralization and Heat Treatment

To evaluate the antibacterial activity of CFS after pH neutralization and heat treatment, a modified method based on the experiments conducted by Divyashree *et al*. [27] was used. CFS was adjusted to pH 6.5 using 5 M NaOH (neutralization), and heated at 100°C for 30 min followed by cooling (heat treatment). After pH adjustment and heat treatment, 100 μl of CFS samples for each pathogen was added to the respective wells of a microplate. Approximately 100 μl of indicator strain diluted to a concentration of about 10^6^ CFU/ml in TSB was inoculated into each well. The microplates were incubated at 37°C for 24 h to allow bacterial growth, which was then visually confirmed and compared.

### Analysis of Organic Acids

High-performance liquid chromatography (HPLC) was performed to analyze the organic acid content produced by CFS. The samples were diluted 10-fold in tertiary distilled water, then centrifuged at 9,950 ×*g* for 10 min and filtered through a 0.22-μm membrane filter. The filtered samples were analyzed using an HPLC system (Ultimate 3000, Dionex, USA) equipped with an Aminex 87H column (300 × 7.8 mm), maintained at 40°C. A mobile phase of 0.01 N H_2_SO_4_ was used at a flow rate of 0.5 ml/min, with samples injected at 10 μl per run. Detection was conducted using a Refractive Index (RI) detector (Shodex RI-101, Japan) and UV detector at 210 nm over a 30 min run time. Organic acids were identified qualitatively by comparing their retention times with standard chromatograms of known organic acids injected into the HPLC system. Quantitative analysis of the organic acids in the samples was performed by calculating the peak areas relative to those of standard organic acids.

### Statistical Analysis

The experimental results were analyzed using GraphPad Prism (version 10.0; GraphPad Software, USA). One-way analysis of variance (ANOVA) was conducted to test the significance (*p* < 0.05) of differences among groups, followed by Tukey's multiple comparisons test. All results are presented as mean ± SD.

## Results

### Antimicrobial Activity of CFS: MIC and MBC Determination

Antibacterial activity against foodborne and soft-rot pathogens was determined for the LAB strains using MIC and MBC values (Table 2). The MIC values ranged from 6.25% to 25% (v/v), whereas the MBC values ranged from 12.5% to 50% (v/v). Except for *E. coli*, WiKim 83 and WiKim 87 showed lower MIC than WiKim 116 and WiKim 117. The lowest MIC was observed for *Y. enterocolitica* and soft-rot pathogens. WiKim 83 exhibited the lowest MBC against *P. carotovorum* KACC 10342. Except for KACC 10371, WiKim 83 and WiKim 87 showed MBC values at least two-fold lower than those of WiKim 116 and WiKim 117. Compared with foodborne pathogens, WiKim 83 and WiKim 87 exhibited equivalent or two-fold lower MBC values against *P. carotovorum*, suggesting stronger antimicrobial activity against soft-rot pathogens than against foodborne pathogens.

### Biocontrol Assay to Determine *B. cereus* Inhibition Using CFS in Salted Kimchi Cabbage

**Fig. 1:**
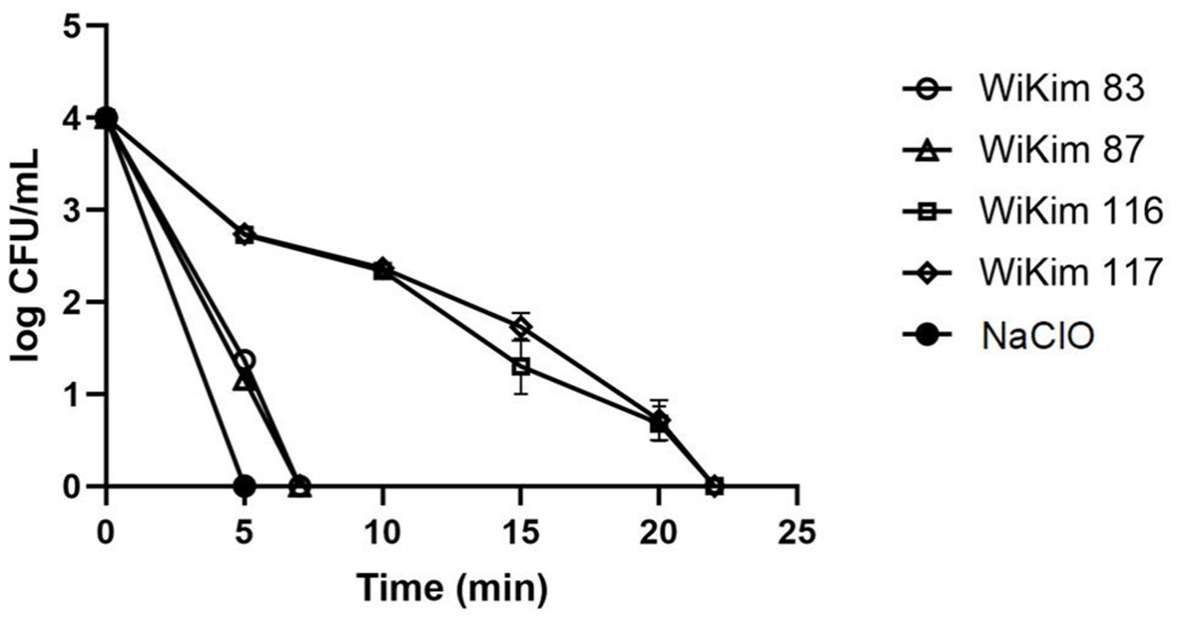
Time-kill assay of the lactic acid bacteria CFS against *B. cereus*. The data are presented as mean ± SD (*n* = 3).

**Fig. 2:**
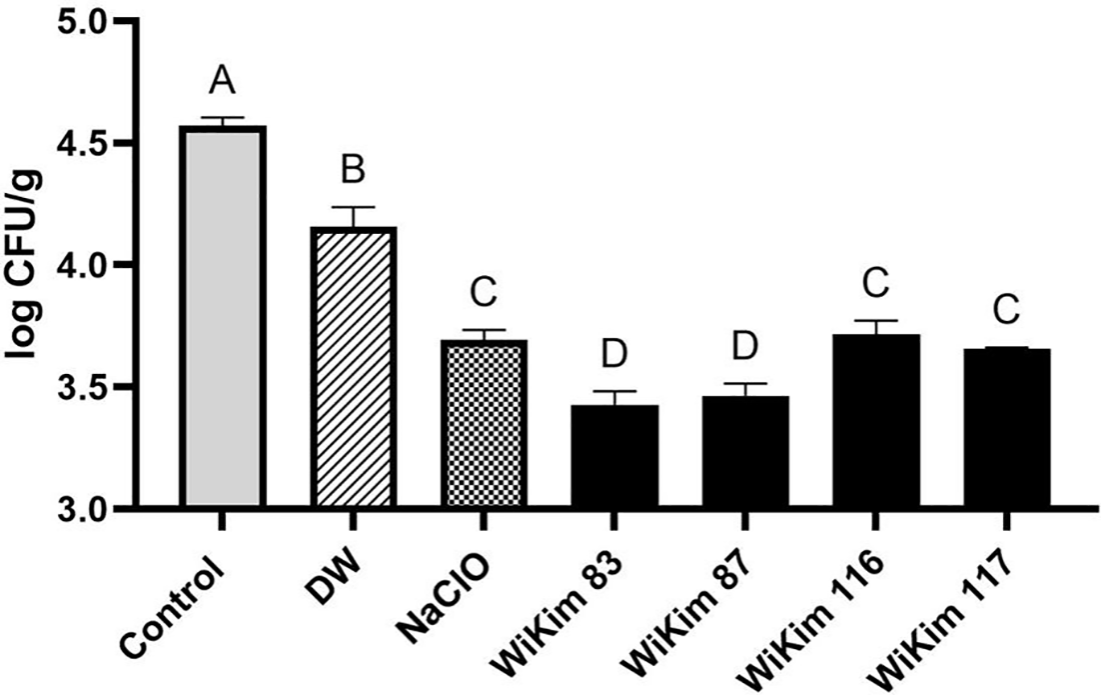
Bacterial growth of *B. cereus* on salted kimchi cabbage treated with lactic acid bacteria CFS, NaClO, and distilled water. The data are presented as mean ± SD (*n* = 3). Statistical significance was determined using one-way ANOVA followed by Tukey's post hoc test; Significant differences between treatments of the salted kimchi cabbage are denoted by different letters (**A-D**), indicating statistical significance (*p* < 0.05).

The bactericidal effects of the four LAB strains on *B. cereus* were assessed over time (Fig. 1). WiKim 83 and WiKim 87 achieved complete inhibition of *B. cereus* within 7 min, whereas WiKim 116 and WiKim 117 required 22 min. The control group (NaClO) exhibited bactericidal activity within 5 min. Salted kimchi cabbage samples inoculated with *B. cereus* were treated with NaClO or CFS, and bacterial inhibition was evaluated (Fig. 2). When comparing the viable counts of *B. cereus* between the CFS-treated and negative control groups, a significant reduction of approximately 1 log CFU/g was observed in the CFS-treated group (*p* < 0.05). Specifically, WiKim 83 and WiKim 87 exhibited significantly stronger inhibitory activity than NaClO (*p* < 0.05), whereas WiKim 116 and WiKim 117 showed control efficacy similar to that of NaClO (*p* < 0.05).

### Biocontrol Assay to Investigate Soft-Rot Disease Bacteria Using CFS on Kimchi Cabbage

**Fig. 3:**
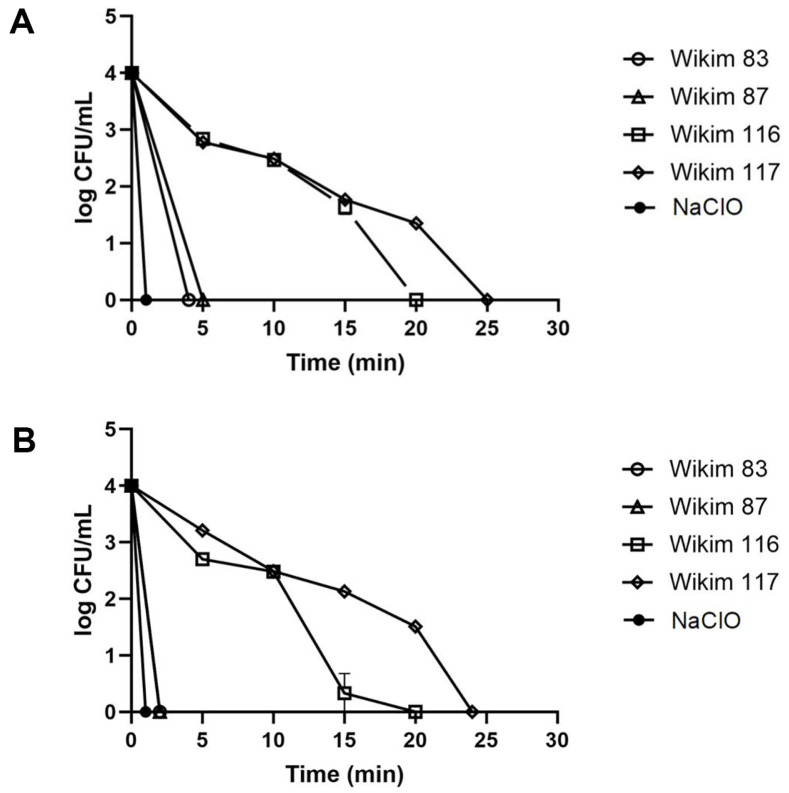
Time-kill assay of the lactic acid bacteria CFS against soft-rot disease bacteria. The data are presented as mean ± SD (*n* = 3). (**A**) *P. carotovorum* KACC 10342 and (**B**) *P. carotovorum* KACC 10371.

**Fig. 4:**
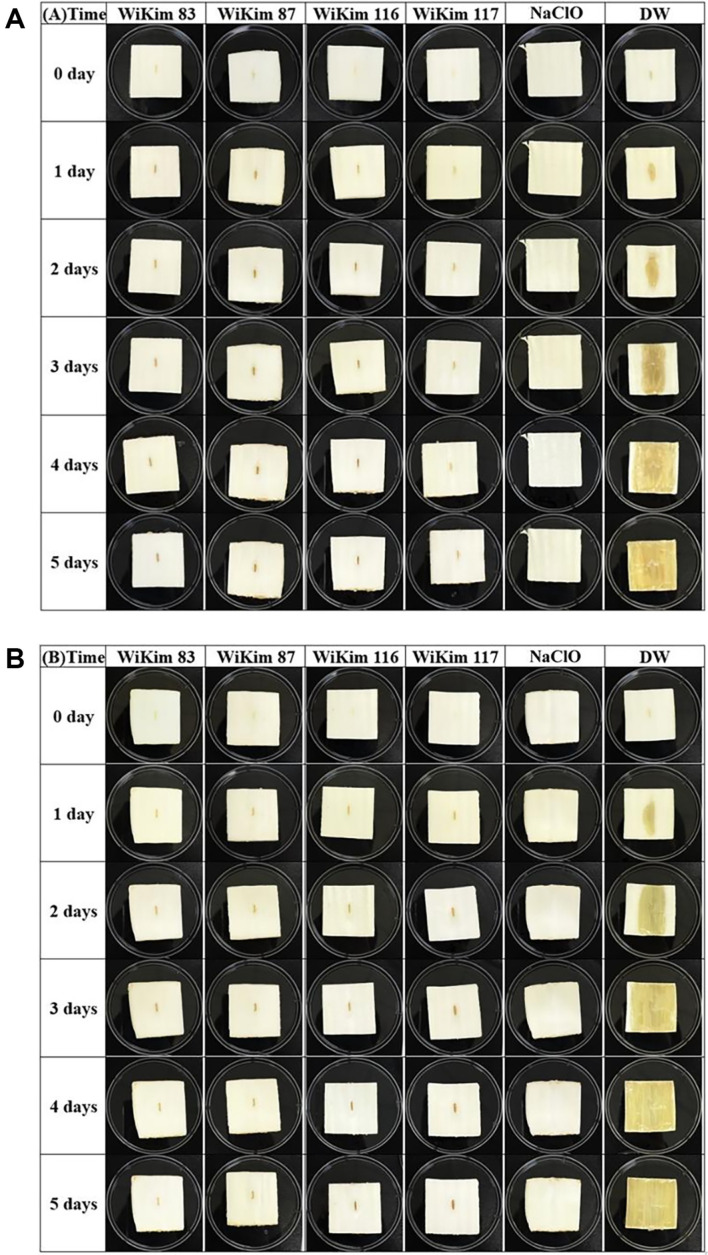
Effect of CFS on soft-rot disease induced by *P. carotovorum* in kimchi cabbage. (**A**) *P. carotovorum* KACC 10342. (**B**) *P. carotovorum* KACC 10371.

The bactericidal activity of CFS against *P. carotovorum* strains KACC 10342 and KACC 10371 was analyzed and revealed time-dependent effects (Fig. 3). For KACC 10342, WiKim 83 exhibited bactericidal effects after 4 min, WiKim 87 after 5 min, WiKim 116 after 20 min, and WiKim 117 after 25 min (Fig. 3A). For KACC 10371, WiKim 83 and WiKim 87 exhibited bactericidal effects after 2 min, WiKim 116 after 20 min, and WiKim 117 after 24 min (Fig. 3B). Based on time-dependent bactericidal analysis, the efficacy of the four CFSs in controlling soft-rot disease in kimchi cabbage was evaluated. By day 5 of cultivation, neither the CFS-treated nor the NaClO-treated group exhibited any signs of soft rot in kimchi cabbage samples treated with KACC 10371 and KACC 10342 (Fig. 4). In contrast, kimchi cabbage treated with distilled water as a negative control showed symptoms of soft rot starting on day 1, with complete manifestations across all parts of the kimchi cabbage by day 5. These results indicate that CFS treatment achieves similar outcomes to NaClO treatment in food washing, effectively controlling bacterial soft rot in kimchi cabbages.

### Effects of pH Neutralization and Heat Treatment

The antimicrobial activities of the four CFSs were evaluated based on pH neutralization and heat treatment conditions (Table 3). The pH values of the CFSs were as follows: WiKim 83 at 3.79 ± 0.01, WiKim 87 at 3.80 ± 0.02, WiKim 116 at 4.12 ± 0.02, and WiKim 117 at 4.10 ± 0.02. Neutralizing the pH to 6.5 resulted in reduced antimicrobial activity against all pathogens tested. In contrast, heat-treated CFSs showed no significant decrease in antimicrobial activity, compared with the untreated control groups, regardless of whether the pH was neutralized. These findings indicate that the CFSs exhibited high stability against heat treatment. Furthermore, the substantial decrease in antimicrobial activity after pH neutralization suggests that the low pH of the CFSs plays a crucial role in their antimicrobial efficacy.

### Analysis of Organic Acids

Table 4 presents the organic acid contents of the four CFSs. Citric acid was the highest in WiKim 87 at 1,679.50 mg/l, whereas it was not detected in WiKim 116 and WiKim 117. Lactic acid was the most abundant in WiKim 83 (16,351.34 mg/l) and the lowest in WiKim 116 (10,902.37 mg/l). Acetic acid was the highest in WiKim 116 (5,370.52 mg/l) and the lowest in WiKim 83 (4,890.76 mg/l). Phenyllactic acid was the highest in WiKim 87 at 75.46 mg/l and the lowest in WiKim 116. The total organic acid content was the highest in WiKim 87 (23,099.52 mg/l) and the lowest in WiKim 116 (16,281.90 mg/l). Total organic acid content was significantly higher in *L. plantarum* strains than in *L. sakei* strains. Lactic and acetic acids were identified as the major organic acids in all four CFSs isolated from LAB.

## Discussion

In this study, the foodborne pathogens used as indicator strains, *B. cereus*, *C. perfringens*, *E. coli*, *L. monocytogenes*, and *Y. enterocolitica*, were among the most relevant pathogens in kimchi, according to the detection standards set by the MFDS. As illustrated by the cases described in the Introduction section, these frequently detected pathogens are capable of inflicting substantial illness in many individuals, making their control during kimchi production essential. While previous studies have reported on the antimicrobial activity of CFS from LAB against foodborne pathogens, the superiority of the activity exhibited by the CFSs used in this study is distinguishable from that in earlier research. For instance, the CFS of *L. plantarum* Cs at a concentration of 25% demonstrated an inhibition rate of less than 50% against *E. coli* [29]. This indicates that WiKim 83 and WiKim 87 were effective in inhibiting *E. coli* at concentrations higher than the MIC values of 12.5%. Additionally, *L. fermentum* was reported to have an MIC of 30% against *E. coli* [30]. Furthermore, the CFS of *Lactococcus* spp. did not achieve bactericidal activity against *L. monocytogenes* at a concentration of 50%, indicating an MBC > 50% [31]. This value was greater than the MBC values observed for WiKim 83 and WiKim 87. Compared with studies on the antimicrobial activity against *C. perfringens* using 17 LAB CFS strains, including *L. plantarum* and *L. sakei* [32], and studies on CFS isolated from sourdough against *Y. enterocolitica* [33], our study used four LAB strains, which consistently exhibited markedly superior inhibitory activity, even when compared with the same species of LAB.

The MFDS specifies a detection limit of < 10^4^ CFU/g for *B. cereus* in kimchi and salted kimchi cabbage. Previous research has indicated that the levels of *B. cereus* in salted kimchi cabbage and cabbage kimchi available on the market range from 0 to 3.40 log in salted kimchi cabbage, and from 0 to 2.32 log in cabbage kimchi [34, 35]. Notably, levels of *B. cereus* reaching 3.4 log have been detected in salted kimchi cabbage [34], which is approaching the detection limit of < 10^4^ CFU/g. These findings indicate that *B. cereus* was detected in salted kimchi cabbage and cabbage kimchi, making it crucial to maintain its levels below the detection limit. Therefore, we set the initial bacterial concentration at 10^4^ CFU/g to assess the effectiveness of washing with CFS in reducing *B. cereus* in salted kimchi cabbage. To replace NaClO (100 ppm), which is typically used in the washing process of salted kimchi cabbage, the application of 5% CFS resulted in a reduction of *B. cereus* comparable to that of NaClO. Specifically, WiKim 83 and WiKim 87 exhibited more effective antimicrobial activity. Previous studies have explored various methods, such as grapefruit seed extract and electrolyzed water, to reduce total microbial counts in salted kimchi cabbages [36, 37]. None of the previous studies sought to develop materials to reduce *B. cereus* in salted kimchi cabbages. Considering the current lack of research on the use of CFS from LAB as an alternative, this study demonstrated its effectiveness in reducing *B. cereus* through direct application. Thus, it is expected to have significant industrial value. Furthermore, these findings can contribute to the identification of effective cleaning methods for reducing *B. cereus* in salted kimchi cabbages.

The antimicrobial effects of CFS against bacterial soft rot in kimchi cabbages were evaluated at various concentrations. Some CFSs exhibited strong antimicrobial activity until diluted to 6.25%. Although numerous qualitative studies have evaluated the antimicrobial activity of LAB CFS against bacterial soft rot in vegetables [38-40], including kimchi cabbage, quantitative research confirming this activity is scarce [41]. The findings from this study, along with their application to food products, are expected to serve as foundational data for understanding antimicrobial activity against soft-rot disease.

In a food test conducted to investigate whether soft-rot pathogens could be controlled within kimchi cabbages, results comparable to those obtained with NaClO treatment were observed. These results showed that while all parts of the kimchi cabbage in the negative control group exhibited rot on day 5, no rot was observed in the kimchi cabbage treated with the four strains of lactic acid bacteria CFS or NaClO. In contrast to the time-based antimicrobial efficacy analysis, which showed a relatively delayed bactericidal effect of WiKim 116 and WiKim 117, the observation that these CFS exhibited activity in kimchi cabbage similar to that of NaClO suggests that the inhibition of soft-rot pathogens by WiKim 116 and WiKim 117 CFS was sustained within kimchi cabbage.

We investigated the effects of pH neutralization and heat treatment on the antimicrobial activity of the CFS, and found that CFS neutralized to pH 6.5 lost its antimicrobial activity. This is consistent with the findings of Arioja *et al*., who reported that the low pH produced by organic acids plays an important role in the antimicrobial activity of CFS against pathogens, such as *E. coli* and *L. monocytogenes* [42].

Similarly, Daranas *et al*. [40] reported that the antimicrobial activity of LAB against soft-rot pathogens can be attributed to pH reduction and lactic acid production. This result is in line with our findings, as we observed that low pH and organic acids are related to antimicrobial effects against soft-rot pathogens. In contrast, the heat-treated CFS showed the same antimicrobial activity, suggesting that the antimicrobial substances present in the four LAB CFS types were heat stable. This implies that the LAB CFS may be beneficial in the food industry because its antimicrobial activity remains effective even under high-temperature conditions. According to Adams *et al*., among the main antimicrobial substances produced by LAB, bacteriocins and hydrogen peroxide contribute secondarily to the stability of fermented foods, whereas organic acid production is a primary factor in lowering food pH [43]. This study supports the concept that organic acids play a significant role in antimicrobial activity against foodborne pathogens and bacterial soft rot.

Therefore, an analysis of the organic acids present in the metabolic products and CFS samples of the LAB was conducted and revealed that the four strains produced organic acids in the following order: lactic acid, acetic acid, and phenyllactic acid. Citric acid production was observed only in the CFS of the Wikim 83 and Wikim 87 strains. The significant differences in organic acid content between WiKim 83 and WiKim 87, compared with WiKim 116 and WiKim 117, suggest varying trends in antimicrobial activity. The stronger antimicrobial activity of WiKim 83 and WiKim 87 compared with that of WiKim 116 and WiKim 117 is likely due to the presence of citric acid, which has antimicrobial properties, as well as a higher content of lactic acid and phenyllactic acid. Compared with existing studies on organic acids in lactic acid bacterial CFS, our analysis revealed that the four lactic acid bacterial strains produced higher levels of lactic acid. Compared with the study by Guimarães *et al*. [44], the organic acid content in these four strains showed relatively lower levels of lactic acid, but similar or higher levels of citric acid, along with greater amounts of acetic acid.

Research into the antimicrobial activity of the abovementioned organic acids is ongoing, particularly concerning their use as food preservatives [45]. Lactic acid is permitted as a food additive under Regulation (EC) No. 1333/2008 and is widely utilized in various food products, whereas acetic acid is commonly employed for the preservation of fruit juices and fresh vegetables [46, 47]. Previous studies using detailed mechanistic analyses demonstrated the strong antimicrobial effects of phenyllactic acid, a metabolite produced by LAB, against *L. monocytogenes* and *E. coli* [48]. Notably, during kimchi fermentation, LAB-produced phenyllactic acid shows significant antimicrobial activity against foodborne pathogens and molds, thereby contributing to the hygiene and safety of kimchi [49, 50]. Citric acid has been confirmed to possess potent antimicrobial effects against foodborne pathogens and shows enhanced efficacy when used in conjunction with acetic acid [51]. Its role as a food preservative for fresh vegetables has also been evaluated previously.

Furthermore, research on the antimicrobial activity of organic acids against soft-rot disease is ongoing. As demonstrated by Zheng *et al*., acetic acid boasts a lower MIC compared to lactic acid against bacterial soft rot in vegetables, indicating a relatively stronger antimicrobial effect [52]. Additionally, studies on the synergistic antimicrobial effects of citric acid in combination with palmarosa oil-loaded nanoemulsions showed that citric acid not only enhanced the stability of the nanoemulsions, but also reduced the MIC more effectively through antimicrobial action mediated by ATP depletion within the cells compared with citric acid used alone [53]. Although research on the antimicrobial mechanisms of LAB metabolic products is limited, it is plausible that citric acid functions in a manner similar to the antimicrobial mechanisms observed in previous studies on *P. carotovorum*. Organic acids produced as metabolic byproducts of LAB have been repeatedly validated for their potential use as food preservatives. Their efficacy as natural food preservatives is well established [54], and research is ongoing to explore their application as natural antimicrobial agents for preventing bacterial soft rot [55, 56].

According to the results of this study, WiKim 83 and WiKim 87 exhibit superior antimicrobial activity against foodborne and soft-rot pathogens, inhibition activity within food, and organic acid production compared to WiKim 116 and WiKim 117. Therefore, WiKim 83 and WiKim 87 are considered more suitable materials for use as food preservatives and disinfectants, respectively. In addition, WiKim 83 and WiKim 87 have previously been studied for their utility as starter cultures in kimchi production, demonstrating both safety and probiotic characteristics, as well as antimicrobial and antioxidant activities [57]. The results of our study suggest that WiKim 83 and WiKim 87 could serve as strong natural food preservatives in fermented foods, such as kimchi, while also serving as disinfectants. Moreover, the results of this study on WiKim 116 and WiKim 117 provide foundational data for further research on their potential applications as natural food preservatives, and can improve food safety and quality.

This study has the potential to establish a basis for antimicrobial activity through further research on the purification and analysis of antimicrobial substances and the mechanisms underlying their antimicrobial action. Subsequent research could validate these findings and provide evidence supporting the antimicrobial activity reported in this study.

## Figures and Tables

**Table 1:** List of lactic acid bacteria and indicator strains used in this study.

Lactic acid bacteria strains	Source	Temperature	Media
*Lactiplantibacillus plantarum* WiKim 83	Kimchi	37°C	MRS^d^
*Lactiplantibacillus plantarum* WiKim 87	Kimchi	37°C	MRS
*Latilactobacillus sakei* WiKim 116	Kimchi	37°C	MRS
*Latilactobacillus sakei* WiKim 117	Kimchi	37°C	MRS
Indicator strains			
*Bacillus cereus* KCCM 40935	KCCM^a^	30°C	TSB^e^
*Clostridium perfringens* ATCC 13125	ATCC^b^	37°C	TSB
*Escherichia coli* KCCM 11234	KCCM	37°C	TSB
*Listeria monocytogenes* KCCM 40307	KCCM	37°C	TSB
*Yersinia enterocolitica* ATCC 23715	ATCC	37°C	TSB
*Pectobacterium carotovorum* KACC 10342	KACC^c^	28°C	TSB
*Pectobacterium carotovorum* KACC 10371	KACC	28°C	TSB

^a^KCCM, Korean Culture Center of Microorganisms

^b^ATCC, American Type Culture Collection

^c^KACC, Korean Agricultural Culture Collection

^d^MRS, De Man Rogosa Sharpe

^e^TSB, tryptic soy broth

**Table 2:** Minimum inhibitory concentration (MIC) and minimal bactericidal concentration (MBC) of the lactic acid bacteria CFS against two types of pathogens.

LAB strain	Foodborne pathogens					Soft-rot pathogens	
	*B. cereus* KCCM 40935	*L. monocytogenes* KCCM 40307	*C. perfringens* ATCC 13125	*E. coli* KCCM 11234	*Y. enterocolitica* ATCC 23715	*P. carotovorum* KACC 10342	*P. carotovorum* KACC 10371
	MIC (%v/v)						
WiKim 83	12.5	12.5	12.5	12.5	6.25	6.25	6.25
WiKim 87	12.5	12.5	12.5	12.5	6.25	6.25	6.25
WiKim 116	25	25	25	12.5	12.5	12.5	12.5
WiKim 117	25	25	25	12.5	12.5	12.5	12.5
	MBC (%v/v)						
WiKim 83	25	50	25	50	25	12.5	25
WiKim 87	25	50	25	50	25	12.5	25
WiKim 116	50	n.d.^a^	50	50	50	25	25
WiKim 117	50	n.d.^a^	50	n.d.^a^	50	25	25

The data represent the results of three replicates.

^a^n.d., not detected

**Table 3:** Antimicrobial activity of the lactic acid bacteria CFS after heat treatment and pH neutralization against pathogens presented as growth inhibition percentage.

LAB strain	Foodborne pathogens					Soft-rot pathogens	
	*B. cereus* KCCM 40935	*L. monocytogenes* KCCM 40307	*C. perfringens* ATCC 13125	*E. coli* KCCM 11234	*Y. enterocolitica* ATCC 23715	*P. carotovorum* KACC 10342	*P. carotovorum* KACC 10371
	Heat treatment						
WiKim 83	+ve^a^	+ve	+ve	+ve	+ve	+ve	+ve
WiKim 87	+ve	+ve	+ve	+ve	+ve	+ve	+ve
WiKim 116	+ve	+ve	+ve	+ve	+ve	+ve	+ve
WiKim 117	+ve	+ve	+ve	+ve	+ve	+ve	+ve
	pH neutralization						
WiKim 83	−ve^b^	−ve	−ve	−ve	−ve	−ve	−ve
WiKim 87	−ve	−ve	−ve	−ve	−ve	−ve	−ve
WiKim 116	−ve	−ve	−ve	−ve	−ve	−ve	−ve
WiKim 117	−ve	−ve	−ve	−ve	−ve	−ve	−ve

The data represents the results of three replicates.

^a^+ve: antibacterial activity observed.

^b^−ve: no antibacterial activity observed.

**Table 4:** Organic acid composition of lactic acid bacteria CFS via HPLC analysis.

LAB strain	Organic acid (mg/l)				
	Citric acid	Lactic acid	Acetic acid	Phenyllactic acid	Total
WiKim 83	1,665.23 ± 64.28^A^	16,351.34 ± 417.23^A^	4,890.76 ± 92.38^A^	71.24 ± 4.61^A^	22,978.57 ± 569.28^A^
WiKim 87	1,679.50 ± 73.26^A^	16,330.83 ± 381.54^A^	5,013.73 ± 74.22^B^	75.46 ± 5.27^A^	23,099.52 ± 387.77^A^
WiKim 116	n.d.^a^	10,902.37 ± 208.46^B^	5,370.52 ± 81.92^A^	9.01 ± 3.86^B^	16,281.90 ± 294.24^B^
WiKim 117	n.d.^a^	11,023.89 ± 241.78^B^	5,276.53 ± 94.19^A^	10.03 ± 4.15^B^	16,310.45 ± 143.44^B^

The data are presented as mean ± SD (*n* = 3). Statistical significance was determined using one-way ANOVA followed by Tukey's post hoc test; Significant differences in organic acids of the same lactic acid bacteria CFS are indicated by different letters (A-B), indicating statistical significance (*p* < 0.05).

^a^n.d., not detected
